# Small Mountainous Rivers Generate High-Frequency Internal Waves in Coastal Ocean

**DOI:** 10.1038/s41598-018-35070-7

**Published:** 2018-11-09

**Authors:** A. A. Osadchiev

**Affiliations:** Shirshov Institute of Oceanology, Russian Academy of Sciences, Moscow, Russia

## Abstract

High-frequency internal waves propagating offshore in small river plumes are regularly observed at satellite imagery in many world regions. In this work we describe a mechanism of generation of these internal waves by discharges of small and rapid rivers inflowing to coastal sea. Friction between river runoff at high velocity and the subjacent sea of one order of magnitude lower velocity causes abrupt deceleration of a freshened flow and increase of its depth, i.e., a hydraulic jump is formed. Transition from supercritical to subcritical flow conditions induces generation of high-frequency internal waves that propagate off a river mouth at a stratified layer between a buoyant river plume and subjacent ambient sea and influence turbulence and mixing at this layer. Basing on *in situ* and satellite data we estimated wavelengths, phase speeds, and frequencies of internal waves generated in small river plumes located off the northeastern coast of the Black Sea. This process is typical for many other world mountainous regions where numerous and closely spaced small and rapid rivers inflow to sea during high discharge periods and can strongly influence, first, structure and dynamics of river plumes and, second, physical, biological, and geochemical processes in adjacent coastal areas.

## Introduction

Internal waves (IW) are common features in deep sea and coastal ocean and significantly influence horizontal and vertical momentum transport, mixing, turbulence, stratification and many related physical, biological, and geochemical processes^1–7^. One of the main sources of IW in the world ocean is interaction of barotropic tidal currents with bathymetry features and generation of baroclinic tide^2,8,9^. IW are also generated by other mechanisms including shear instability, atmospheric forcing, coastal upwelling, and gravity currents^10,11^.

Nash and Moum described a mechanism of generation of IW in coastal ocean by a stratified current formed by freshwater discharge to tidal sea^12^. Spreading of a buoyant river plume during ebb tide can cause surface convergence of horizontal velocity at the frontal zone between a plume and ambient sea. Subsequent downward displacement of buoyant water at the plume front converts kinetic energy of a plume to potential energy and cause generation of packets of IW which propagate from the plume front offshore to ambient sea. A number of works thoroughly studied many aspects of this mechanism at the large Columbia River plume including its transition from supercritical to subcritical regimes, generation and propagation of non-linear IW, and influence of IW on local ecosystem^13–17^.

This study describes a new mechanism of generation of high-frequency IW in coastal ocean by freshwater discharge, which to the extent of our knowledge was not addressed before. A river with small discharge rate and high flow velocity runoffs to coastal sea and forms a hydraulic jump in vicinity of its mouth. This process effectively transforms kinetic energy of river flow to potential energy and cause generation of high-frequency IW. The main differences between this mechanism and the mechanism described by Nash and Moum are as follows. First, IW are generated by runoffs of small rivers, which significantly increases the number of world rivers which discharge potentially can generate IW. Second, generation of IW is not related to tidal forcing and can occur outside ebb tide. Third, IW are emerged from a small area in vicinity of a river mouth, which location is relatively stable as compared to a highly variable plume front.

## Results

### Study region

**Figure 1:**
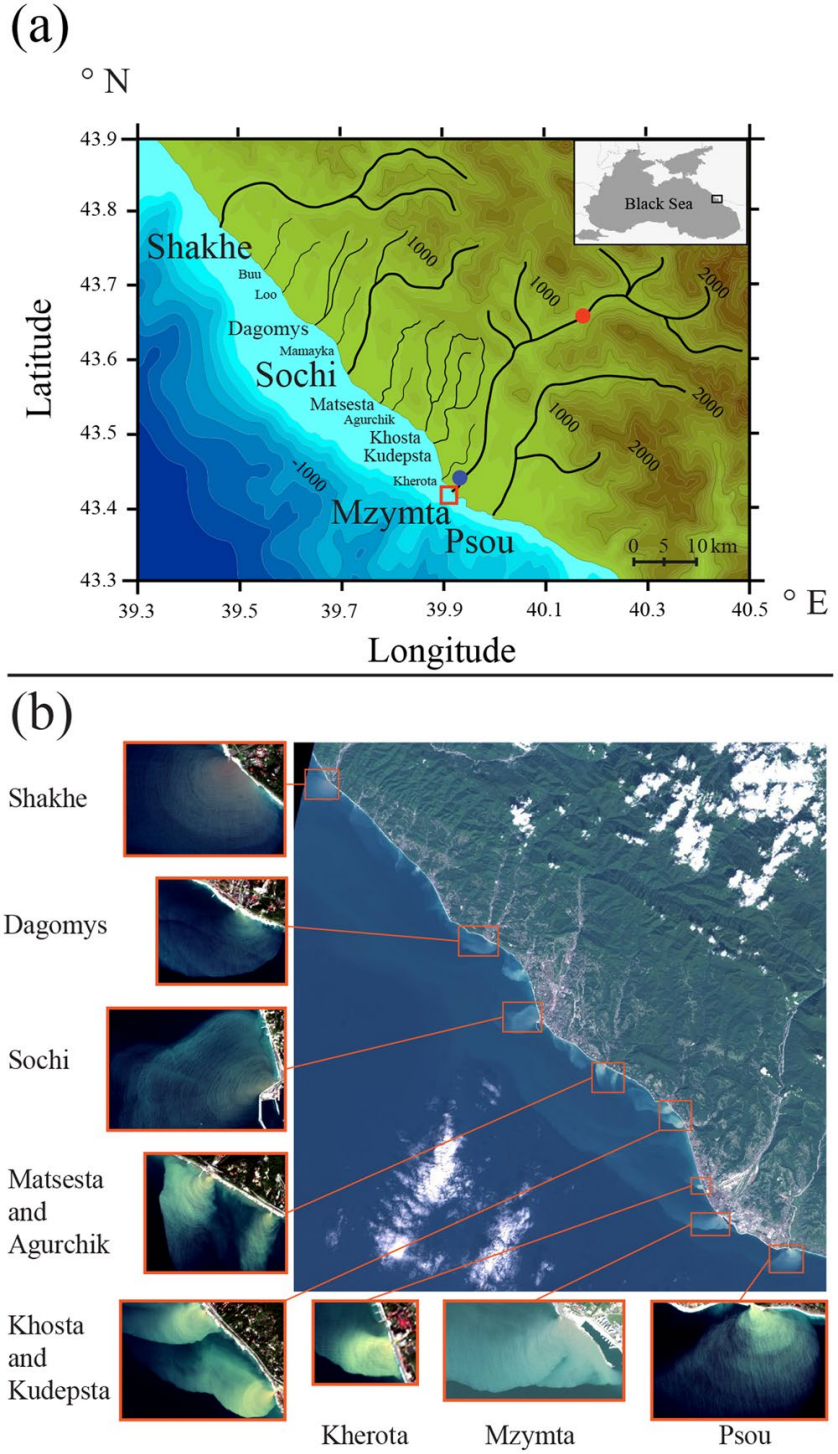
Bathymetry, topography, and main rivers of the study region; location of the area of field works at the Mzymta plume (red rectangle); location of the Adler airport meteorological station (blue circle) and the Krasnaya Polyana gauge station (red circle) (**a**). Sentinel-2 ocean color composite from 30 September 2016 indicating IW generated by small rivers of the study region (**b**). Satellite imagery was created using SNAP software package (version 5.0.8), http://step.esa.int/main/toolboxes/snap/.

This work is focused on high-frequency IW generated by discharges of numerous small mountainous rivers that inflow to the northeastern part of the Black Sea (Fig. 1a). Mean annual runoffs of these rivers are less than 50 m^3^/s; however, their daily discharges are 2–3 times greater during spring freshet periods and 1–3 orders of magnitude greater during regular rain-induced flash flooding events^18,19^. Drainage basins of these rivers are located in steep and narrow gorges of the Greater Caucasus Range. During freshet and flooding periods flow velocities of these rivers exceed 1.5–2 m/s, while their widths do not significantly change in response to discharge fluctuations.

Horizontal and vertical spatial scales of the buoyant plumes formed by the rivers of the study region are 1–10 km and 0.1–4 m, respectively (except short-term flooding discharge conditions); therefore, their structure and dynamics are characterized by high variability in response to external forcing conditions^20–23^. The shelf at the study area is very steep and narrow; the 100 m isobath is located 2–15 km far from the coast. As a result, liftoff points of river plumes are located in river mouths and river plumes do not exhibit friction with sea bottom. Ambient ocean salinity at the study region is equal to 17–19^24,25^. Tidal amplitudes at the Black Sea are less than 10 cm, at its northeastern part they do not exceed 6 cm; thus, tidal circulation is very low at the study area^26,27^.

### Satellite observations of high-frequency IW in small river plumes

Landsat 8 and Sentinel-2 ocean color composites regularly reveal surface expressions of high-frequency IW propagating in small river plumes off the northeastern shore of the Black Sea (Fig. 1b). Sources of these IW are small areas (100–200 m long and 25–100 m wide) adjacent to river mouths and elongated in directions of river inflows. IW propagate radially from these areas through river plumes towards their fronts with ambient ocean. Wavelengths of IW detected at satellite imagery in different river plumes of the study area were spanned between 30 and 60 m. If area of a river plume is large enough, surface manifestations of IW are distinctly observed near a river mouth and then their intensity steadily decreases with increase of distance to their source. Finally, IW are not visible at optical satellite imagery starting from a certain distance from a river mouth, which vary from 1 to 5 km for different river plumes and different observation periods (Fig. 1b). If spatial scales of a river plume are less than decay distance of IW, they abruptly dissipate on lateral boundary of a river plume. Surface expressions of IW are not observed outside river plumes (Fig. 1b).

Landsat 8 and Sentinel-2 missions provide global coverage with a 5-day repeat cycle of Sentinel-2 and a 16-day repeat cycle of Landsat 8. Some intersections of orbital paths of Sentinel-2 and Landsat 8 are passed by these satellites with small time intervals (5–8 minutes).

As a result, the study area is regularly observed by Sentinel-2 and Landsat 8 sensors nearly simultaneously and with similar view angles^28^ that provide opportunity for retrieving phase speeds of individual IW in the following way. First, position of a specific individual IW is identified on the first image. Then this specific IW is identified on the second image taken several minutes later. Thus, the distance travelled by this IW is obtained by comparing its positions on both images. Phase speed of this IW is directly calculated as a ratio between this distance and time period between satellite observations. As a result, phase speeds and, therefore, periods of individual IW can be reconstructed from near simultaneous satellite imagery. Four pairs of near simultaneous Landsat 8 and Sentinel-2 ocean color composites obtained on 23 April and 30 September 2016, 9 March and 10 September 2017 with small time intervals (5–8 minutes) were analyzed. The obtained ranges of phase speeds and periods of IW calculated for these days at multiple river plumes of the study region were equal to 0.45–0.65 m/s and 65–90 s, respectively.

### IW generated by the Mzymta River discharge

**Figure 2:**
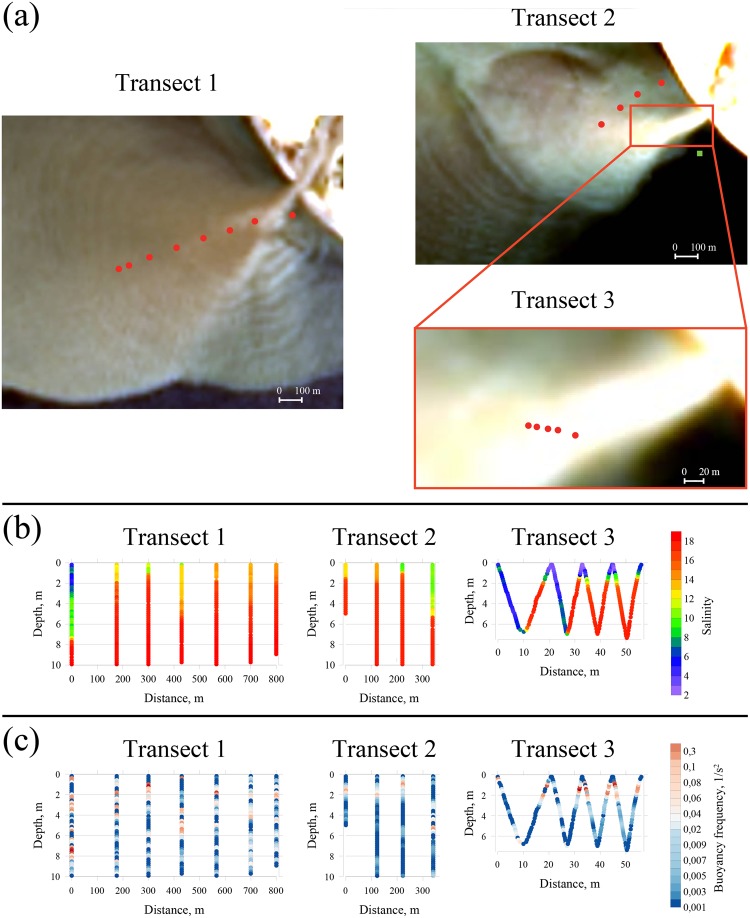
Location of the hydrological stations (red circles) and the current station (green square at transect 2) during the field works on 21 May 2013 (transect 1, left) and 29 May 2014 (transect 2 and transect 3, right) shown on Landsat 8 ocean color composites from 17 May 2013 (left) and 29 May 2014 (right) (**a**). Vertical salinity (**b**) and squared Brunt–Väisälä frequency (*N*^2^) (**c**) profiles obtained at hydrological stations along the transects. Satellite imagery was created using SeaDAS software package ( version 7.3.1), https://seadas.gsfc.nasa.gov/.

Measurements of vertical thermohaline structure at the Mzymta plume were performed along three cross-shore transects on 21 May 2013 and 29 May 2014 (Fig. 2a). In 2013 7 hydrological stations were located along a 800 m long transect (transect 1), the most shoreward station was situated 160 m offshore from the Mzymta mouth (Fig. 2a, left). In 2014 hydrological stations were located along two transects (transect 2 and transect 3) (Fig. 2a, right). The transect 2 (340 m, 4 stations) was shifted 100–150 m northwestward from the Mzymta mouth. The transect 3 was short (56 m long), its most shoreward point was situated 90 m offshore from the Mzymta mouth. The CTD instrument was towed along this transect and was 4 times lowered and raised between the sea surface and the depth of 7–8 m.

Moderate (2–3 m/s) eastward winds were dominating during both periods of field work, while discharge of the Mzymta River was 75 and 64 m^3^/s during these days in 2013 and 2014, respectively. IW in the Mzymta plume were observed at the Landsat 8 satellite images taken on 17 May 2013, i.e., four days before *in situ* measurements along the transect 1, and at 08:01 on 29 May 2014, i.e., several hours before the *in situ* measurements along the transect 2 and transect 3, which were performed at 09:20–10:40 and 11:27–11:29, respectively.

Transect 1 and transect 2 were located close to the Mzymta mouth in the area where propagation of IW is regularly detected by satellite observations. Depth of the boundary between the Mzymta plume and the subjacent sea (defined by 16 isohaline) varied between 1 and 7 m at the transect 1 and between 1 and 5 m at the transect 2 (Fig. 2b). Vertical salinity distributions and plume depths strongly and irregularly changed at distances ~100 m between neighboring stations and showed no dependence on distance to the Mzymta mouth. These features are presumed to be caused by propagation of high frequency IW in the Mzymta plume. They were detected because time intervals between measurements at neighboring stations (10–30 minutes) were significantly greater than periods of IW (65–90 s); therefore, vertical measurements on hydrological stations were performed at random phases of IW. As a result, these data is insufficient to calculate amplitudes of these IW, however, they are estimated to be greater than 6 and 4 m in 2013 and 2014, respectively.

The transect 3 was located within the transition area between the jet-like river inflow (near-field plume) and the outer part of the Mzymta plume, where plume dynamics is governed by external forcing (far-field plume). This transition area appears at satellite imagery to be the source of IW and its thermohaline structure was revealed by CTD measurements with high spatial resolution (7 m). Low-saline waters (3–6) were observed from the surface to the depth of 7 m along the first 9 meters from the beginning of the transect 3, followed by abrupt (2 m wide) transition to high-saline waters (14–18) registered at the depth of 2–7 m along 11–18 m of the transect 3 (Fig. 2b). The same structure was observed along 19–27 m of this transect with sharp gradient (1 m wide) at the depth of 7.5 m between the freshened plume (2–8) and the ambient sea (16–18). These vertical penetrations of freshened water to the depth of 7–7.5 m are anomalous for the Mzymta plume, as well as for plumes formed by rivers with small discharge rates in other world coastal regions. Stratification at the boundary between the Mzymta plume and the subjacent ocean revealed along the transect 3 is among the largest reported in coastal ocean. Values of squared Brunt–Väisälä frequency (*N*^2^) at the individual vertical profiles near the transition area were equal to 0.3–0.6 1/s^2^ (Fig. 2c).

Average depth and width of the Mzymta River mouth were approximately 1 and 40 m during the periods of field work, therefore inflow velocity of the Mzymta River was 1.5–2 m/s. Ambient sea circulation measured 180 m offshore the Mzymta mouth during the second field survey was 2 orders of magnitude less intense (Fig. 2a). Average and maximal current velocities at the depth of 8 m deep were equal to 0.06 and 0.1 m/s, while at the sea bottom layer they were 0.01 and 0.03 m/s. Large velocity shear between the inflowing river discharge and the ambient sea resulted in strong friction between them and, therefore, caused abrupt deceleration of a freshened jet and its downward intrusion, i.e., formation of a hydraulic jump^29^. *In situ* measurements presented at Fig. 2 show distinct difference in vertical thermohaline structure between the source area of IW and area of propagation of IW within the plume and confirm presence of a hydraulic jump in vicinity of a river mouth. The resulting anomalously deep penetrations of low saline water followed by sharp gradients with ambient sea were detected at the first half of the transect 3.

**Figure 3:**
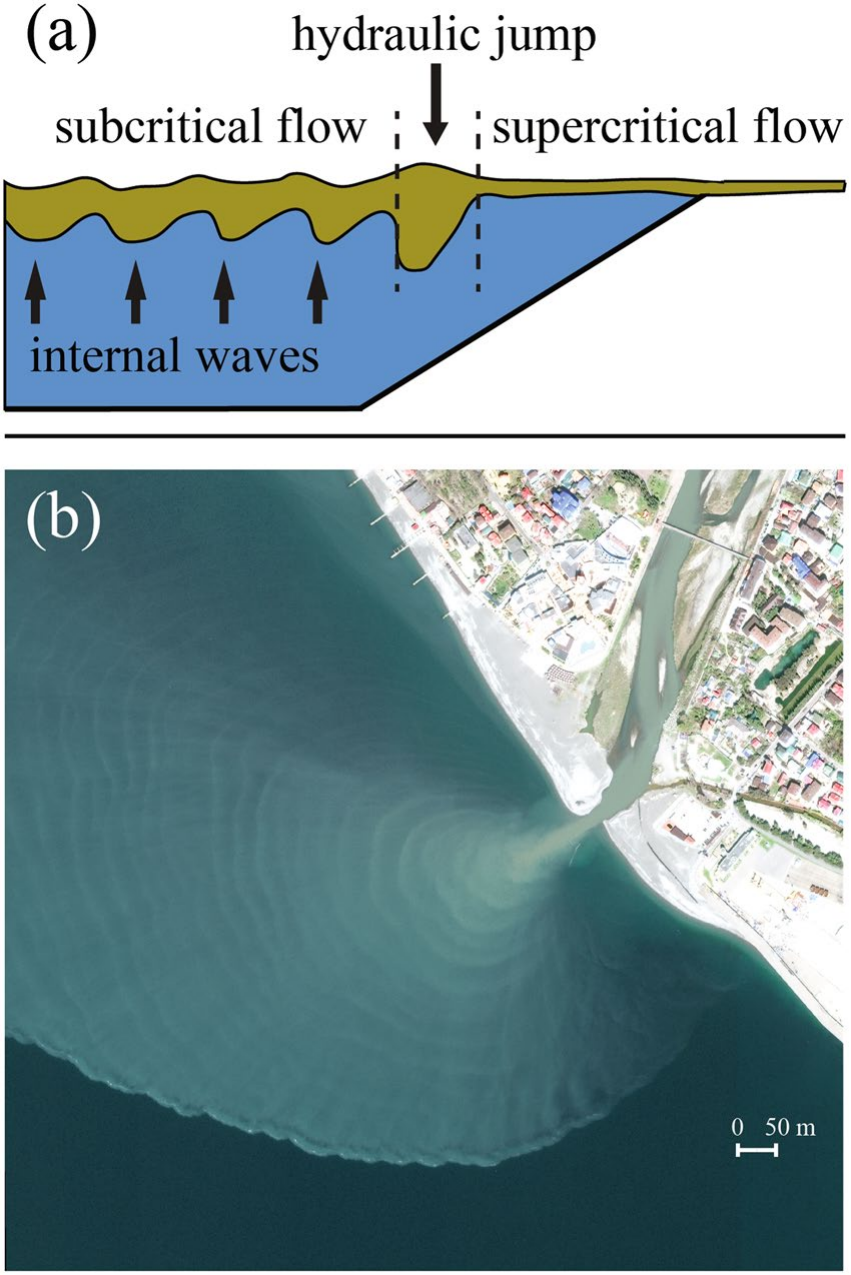
Schematic of formation of a hydraulic jump and generation of IW by river discharge (**a**). WorldView-3 ocean color composite of the Mzymta plume from 4 April 2017 illustrating formation and propagation of IW with high spatial resolution (**b**). This figure is not covered by the CC BY licence. All rights reserved, used with permission. Image © 2017 DigitalGlobe, Inc.

As it was mentioned before, the transect 3 crossed the source area of IW visible at satellite imagery. The frontal Froude number *F* = *u*/*c*, where *u* is the flow velocity and *c* is the internal wave speed is a critical parameter governing generation of IW^12–15^. According to satellite data, *c* is estimated equal to 0.45–0.65 m/s, while *u* in the river mouth was 1.5–2 m/s. The plume liftoff point is located in the river mouth due to steep sea bottom at the study region, river inflow is not decelerated by friction with sea bottom and the freshened flow is strongly supercritical in vicinity of the river mouth (*F* > 1). Therefore, a hydraulic jump formed as a result of the transition of the freshened flow from the supercritical near-field plume to the subcritical far-field plume generated IW, which were detected by satellite imagery and *in situ* measurements. A number of previous studies addressed generation of IW by a hydraulic jump in a two-layered fluid^30,31^. A schematic diagram of this process occurring in a river plume is shown in Fig. 3.

IW propagate seaward in a strongly stratified layer between the freshened far-field Mzymta plume and the subjacent saline sea water. If the Mzymta plume is small and IW do not decay before they reach its lateral boundary, they abruptly dissipate at this boundary, due to relatively low thermal-induced stratification in the ambient sea in the study area^24^. This feature is distinctly visible at Fig. 3b, which shows presence of IW at the northern part of the Mzymta plume till its sharp northern lateral boundary and absence of IW right off this boundary at the adjacent sea. As a result, energy of IW is transformed to turbulence and mixing at the layer between the river plume and the subjacent sea, but is not transported outside the plume area and do not significantly influence mixing in the ambient sea.

### Influence of external forcing conditions on generation of IW

Joint analysis of Sentinel-2 and Landsat 8 satellite data, on the one hand, and wind and river discharge data, on the other hand, during 2013–2017 provided opportunity to reveal dependence between the process of generation of IW in river plumes and external forcing conditions. First, IW were not observed if river discharge was smaller than 50 m^3^/s and were observed during the majority of days when river discharge exceeded 50 m^3^/s. We presume that if discharge rate and, therefore, flow velocity, is lower than a certain threshold velocity shear between a river inflow and ambient ocean is not large enough to form a hydraulic jump and generate IW. In particular, this situation is observed if discharge rate of the Mzymta River is smaller than 50 m^3^/s. However, during a number of days with high discharge conditions IW were not observed.

Analysis of meteorological data showed that generation of IW also depends on wind forcing that govern spreading and mixing of river plumes and, thus, determine areas of propagation and dissipation of IW. First, IW are not generated during periods of high discharge under strong winds (>10 m/s), which cause intense mixing and rapid dissipation of river plumes at the study region. In this case stratification in the coastal sea is not strong enough for propagation of IW^32^. Second, IW are not generated under moderate upwelling (>6 m/s) and downwelling (>4 m/s) wind forcing, presumably, because these winds arrest a river plume along the shore and hinder propagation of IW due to large angle between a river inflow and a far-field plume.

**Figure 4:**
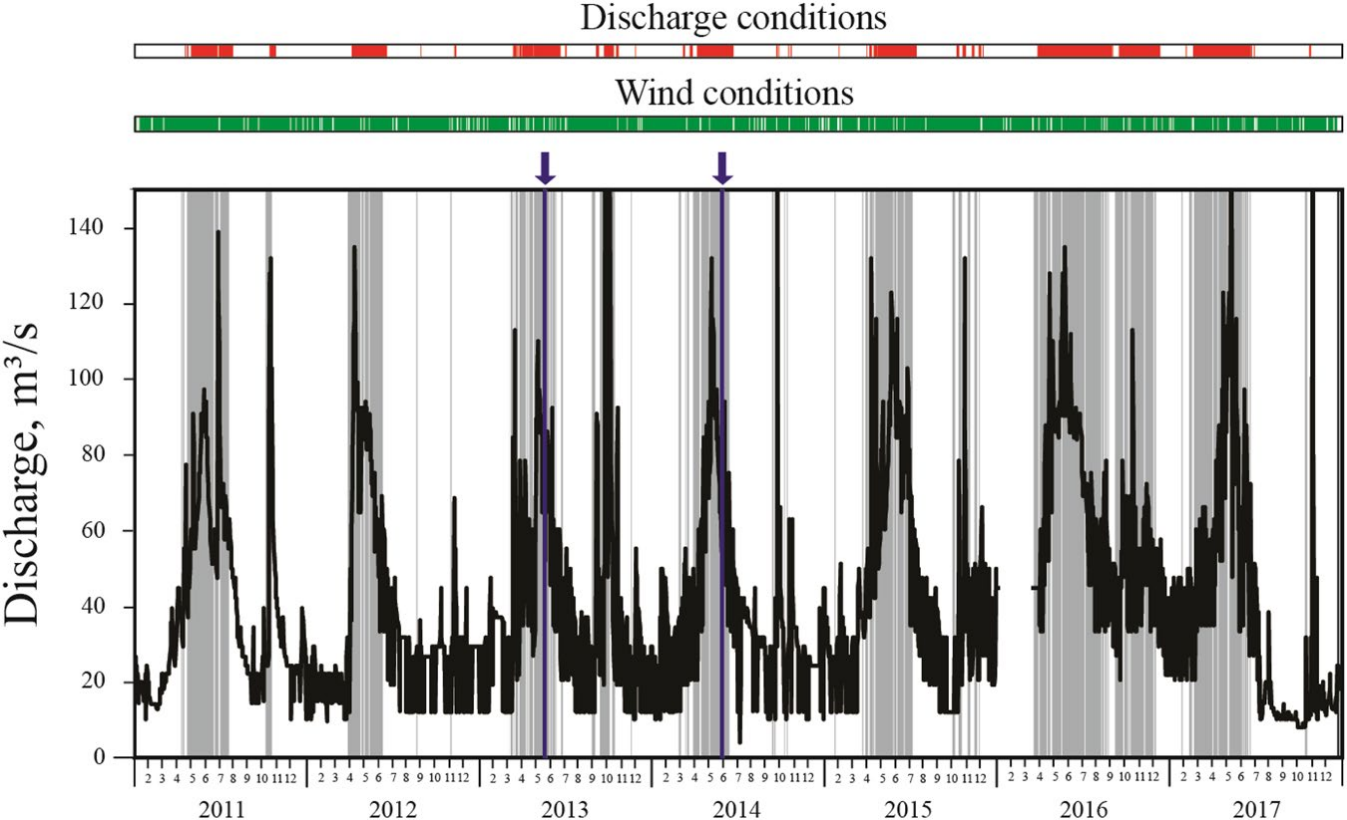
Mzymta River discharge (black line) in 2011–2017, periods of discharge (exceeding 50 m^3^/s, red bands) and wind forcing (green bands) favorable for generation of IW. The resulting periods of external conditions favorable for generation of IW are indicated by grey bands, periods of *in situ* measurements are indicated by blue arrows and lines.

Wind and discharge conditions favorable for generation of IW described above regularly occur at the study region. Basing on the data obtained from the gauge and meteorological stations we identified periods of favorable conditions for generation of IW by the Mzymta River discharge in 2011–2017 (Fig. 4). These periods account for over 80% of days during spring freshets in April – June, while their mean annual duration is estimated as 125 days or approximately one third of the whole year. However, this duration shows significant inter-annual variability from 21% in 2012 to 63% in 2016. IW are generated during 94% of days with high discharge rate; therefore, low river discharge rate is the main limiting factor for generation of IW (Fig. 4).

## Discussion

River runoff forms a hydraulic jump in coastal sea under certain conditions defined by properties of a river flow, ambient sea water, and a local topography. First, a river current has to be fast enough to form a supercritical freshened flow in vicinity of a river mouth. On the other hand, kinetic energy of a freshened flow has to be low enough to be inhibited by friction with ambient sea along a strongly stratified bottom boundary of a river plume^33,34^. This condition is satisfied if river discharge rate is low, i.e., river is rapid, but small. Second, salinity of ambient sea has to be high enough to provide effective conversion of kinetic energy of an inflowing river current to potential energy of a hydraulic jump, which depends on salinity anomaly between a river inflow and ambient sea^35^. This condition is satisfied if coastal circulation hinders freshwater accumulation in vicinity of a river mouth and maintains renewal of sea water in this area. Third, sea depth in vicinity of a river mouth has to be significantly greater than vertical scale of a near-field river plume. In this case, a plume liftoff point is located near a river mouth and a river plume does not interact with sea bottom, so its horizontal velocity convergence has enough space in vertical direction to form a hydraulic jump.

The conditions of formation of hydraulic jumps and generation of IW listed above are typical for many world coastal regions where small and rapid rivers inflow from land with steep topography into sea with steep bathymetry. Recent *in situ* measurements of IW waves generated by a hydraulic jump formed by river discharge in Doubtful Sound, New Zealand were reported by McPherson *et al*.^36^. Generation of IW is also regularly observed by satellite imagery in regions, where rivers discharge to sea from mountain valleys, including the Pacific coasts of Mexico, Peru, and Chile; Taiwan, New Guinea, New Zealand, Western Balkans, Western Caucasus. These rivers often have relatively small discharges due to small areas of their drainage basins in mountain valleys; however, steep slopes of valleys result in high velocities of their flows. Steep bathymetry typical for such regions favors effective renewal of sea water by coastal circulation in deep areas near river mouths. Many of such regions are characterized by seasons of intense rainfalls, which cause flash flooding events on small rivers^37–39^. The resulting simultaneous generation of high-frequency IW from multiple and closely spaced river mouths was registered in several of the mountainous regions listed above. Satellite images illustrating this process observed on 30 September 2016 by Sentinel-2 along the northeastern part of the Black Sea are shown in Fig. 1. On the other hand, for many small rivers in these coastal regions, which topography and bathymetry are favorable for formation of hydraulic jumps, generation of IW is observed irregularly or not observed at all. Low river current speed and, therefore, low discharge rate is the main limiting factor of generation of IW by the Mzymta River, which is, presumably, the case for the rivers in other coastal regions.

The processes of generation, propagation, and dissipation of IW described in this study effectively transforms kinetic energy of a river flow to turbulence in bottom and lateral frontal zones of a river plume, thus, increase mixing in these areas and reduce volume of freshwater contained within a river plume. This pattern of energy transform observed for small rivers at the northeastern part of the Black Sea is significantly different from that typical for larger rivers and/or rivers with less rapid currents, which discharges form recirculating bulges in vicinity of river mouths instead of hydraulic jumps^40–43^. In this case, river kinetic energy is transformed to kinetic energy of an anticyclonic flow within the bulge and potential energy of pressure gradient between a bulge and ambient sea. As a result, river kinetic energy favors accumulation of freshwater in a bulge and reduces mixing between a river plume and ambient sea^44–46^. Thus, generation of IW in river plumes formed by small and rapid rivers can result in their significantly different structure and spreading and mixing dynamics as compared to other river plumes.

## Methods

### *In situ* data

The *in situ* data used in this study were obtained during field surveys on 21 May 2013 and 29 May 2014 at the spreading area of the Mzymta plume (Fig. 1a). Vertical thermohaline structure at the study area was measured at 7 in 2013 and 9 in 2014 hydrological stations by a CTD instrument (*SeaBird SBE19plus*) from surface to a depth of 7–10 m and averaged over 0.2 m depth layers. Measurements of coastal circulation were performed at 1 mooring station in 2014 by an acoustic Doppler current meter (*Nortek Aquadopp*) located at the depth of 8 m (sampling period 1 min) and by a mechanical current meter (*SeaHorse* equipped by *Lowell MAT Logger*) located at the sea bottom (sampling period 10 min). Measurements of the Mzymta discharge and local wind forcing were performed at the Krasnaya Polyana gauge station (daily) and the Adler airport meteorological station (sampling period 3 hours) (Fig. 1a) and were provided by the Federal Service for Hydrometeorology and Environmental Monitoring of Russia.

### Satellite data

The satellite data used in this study consist of ocean color composites from Sentinel-2 MSI (10 m spatial resolution) and Landsat 8 OLI (30 m spatial resolution) sensors. The Landsat 8 Surface Reflectance Level-2 products were downloaded from the United States Geological Survey web repository (http://earthexplorer.usgs.gov). The Sentinel-2 Level-1C products were downloaded from the Copernicus Open Access Hub (https://scihub.copernicus.eu/). Atmospheric correction was applied to these products using Sen2Сor module version 2.2.1 within the Sentinel-2 Toolbox (S2TBX), Sentinel Application Platform (SNAP) version 5.0.7. Due to misregistration issue of Sentinel-2 and Landsat 8 imagery reported by Storey *et al*.^47^. the pairs of ocean color composites used in this study were manually reprojected to obtain good alignment between them.

## Data Availability

The Landsat 8 Surface Reflectance Level-2 products were downloaded from the United States Geological Survey web repository http://earthexplorer.usgs.gov (available after registration). The Sentinel-2 Level-1C products were downloaded from the Copernicus Open Access Hub https://scihub.copernicus.eu/ (available after registration). The river discharge and wind data were downloaded from the Federal Service for Hydrometeorology and Environmental Monitoring of Russia repositories http://gis.vodinfo.ru/ (available after registration) and https://rp5.ru/.
